# A case report of severe tuberous sclerosis complex detected in utero and linked to a novel duplication in the *TSC2* gene

**DOI:** 10.1186/s12883-020-01905-y

**Published:** 2020-09-01

**Authors:** Valérie Mongrain, Nicolaas H. van Doesburg, Françoise Rypens, Catherine Fallet-Bianco, Justine Maassen, Julien Dufort-Gervais, Lucie Côté, Philippe Major

**Affiliations:** 1 Department of Neuroscience, Université de Montréal, Montreal, QC Canada; 2 Center for Advanced Research in Sleep Medicine, Recherche CIUSSS-NIM (site Hôpital du Sacré-Coeur de Montréal), 5400 Gouin West blvd., Montreal, QC H4J1C5 Canada; 3 Centre intégré de diagnostic prénatal (CIDP) and Pediatric Neurology Service, Centre Hospitalier Universitaire (CHU) Ste-Justine, 3175 Chemin de la Côte-Sainte-Catherine, Montréal, QC H3T1C5 Canada

**Keywords:** Ultrasound, Magnetic resonance imaging (MRI), Autopsy, Tuberous sclerosis complex 2 (*TSC2*), *TSC2* c.5169dupA, Case report

## Abstract

**Background:**

Disease severity is tremendously variable in tuberous sclerosis complex (TSC). In contrast with the detailed guidelines available for TSC diagnosis and management, clinical practice lacks adequate tools to evaluate the prognosis, especially in the case of in utero diagnosis. In addition, the correlation between genotypes and phenotypes remains a challenge, in part due to the large number of mutations linked to TSC. In this report, we describe a case of severe TSC diagnosed in utero and associated with a specific mutation in the gene tuberous sclerosis complex 2 (*TSC2*).

**Case presentation:**

A mother was referred for a thorough investigation following the observation by ultrasound of cardiac abnormalities in her fetus. The mother was healthy and reported frequent, intense and long-lasting hiccups/spasms in the fetus. The fetus of gestational age 33 weeks and 4 days was found to have multiple cardiac tumors with cardiac ultrasound. Brain magnetic resonance imaging (MRI) performed in utero revealed the presence of sub-ependymal nodules and of abnormal signals disseminated in the white matter, in the cerebral cortex and in the cerebellum. Following diagnosis of definite TSC, pregnancy interruption was chosen by the parents. Genetic testing of the fetus exposed a duplication in exon 41 of *TSC2* (c.5169dupA), which was absent in the parents. The autopsy ascertained the high severity of brain damage characterized by an extensive disorganisation of white and grey matter in most cerebral lobes.

**Conclusions:**

This case presentation is the first to depict the association between a de novo *TSC2* c.5169dupA and multi-organ manifestation together with indications of a particularly high disease severity. This report can help physicians to perform early clinical diagnosis of TSC and to evaluate the prognosis.

## Background

Tuberous sclerosis complex (TSC) is a rare genetic disease with a reported incidence between 1/6,000 and 1/18,000 affected individuals in the population [1–4]. It can be a devastating condition and is associated with the development of benign tumors/lesions in multiple organs [5–7]. Tumors, which can interfere with normal organ development and/or function, are indeed generally observed in the brain, heart, skin, kidneys and lungs [6–8]. However, the number of organs affected as well as the severity of tumors within an organ is extremely variable [9, 10]. As a result, symptoms of the disease are enormously diverse and variable.

The predominant neurological and psychiatric manifestations of TSC include epilepsy, intellectual disability, autism, hyperactivity and mood disorders [11–13]. Epilepsy can manifest as infantile spasms and is present in up to 90% of TSC patients [5, 6, 9]. It has been proposed as a major factor contributing to intellectual disability [13, 14]. In parallel, sleep disturbances in children with TSC were shown to associate with poorer health [15]. These manifestations can result from the different types of specific brain lesion characteristic of TSC including subependymal nodules, cortical tubers and giant cell astrocytomas [7, 8, 16]. In general, the extent of brain lesions positively associates with neurological and psychiatric manifestations of TSC [7, 17, 18], but the severity of manifestations remains difficult to predict [9, 19]. Outside the brain, the main manifestations of TSC are skin hypomelanotic macules and angiofibromatomas, cardiac rhabdomyomas, renal angiomyolipomas, retinal hamartomas, and lung lymphangioleiomyomatomas [5, 6, 8]. However, the identification of these manifestations is limited in the context of early life, especially in cases of in utero diagnosis.

In approximately 85% of cases, TSC has been associated with genetic variations in two specific genes: tuberous sclerosis complex 1 (*TSC1*) and 2 (*TSC2*) [7, 8, 20]. Hundreds of mutations have been reported to occur in these genes in TSC, including nonsense and missense mutations, insertions and deletions [8, 9]. The type and location of *TSC1* and *TSC2* mutations seem to affect phenotype, but clear genotype-phenotype correlations are difficult to establish [8, 21]. In fact, specific variants are rarely associated with precise phenotypes [9, 22, 23] and with few exceptions [21, 24], most mutations have not been characterized with functional studies. Poor genotype-phenotype correlation could originate from genetic mosaicism or from the need for a second genetic event as proposed in the two-hit model [25]. Thus, genetic analysis of TSC is importantly helpful in diagnosis [8, 26], but is not necessarily informative of disease severity.

Given the very high variability in the diverse manifestations of the disease [9, 10], the diagnosis of TSC as well as the establishment of a severity prognosis remains a challenge, especially when clinical signs of the disease are observed early in life [26]. Furthermore, a major challenge remains concerning the guidance of parents with regards to a decision about pregnancy continuation when TSC signs are present before birth, even when a definite diagnosis is made, because of the absence of clear tool sets to evaluate disease severity. We here describe one case of definite TSC, diagnosed at fetal age using ultrasound and magnetic resonance imaging, and further characterized using genetic and post-mortem biometry and microscopy, which associated with multi-organ manifestations and indications of a high disease severity. Our observations can be used to guide physicians to perform early clinical diagnosis of TSC and to evaluate the prognosis.

## Case presentation

### Parental and clinical history

The mother was a 40-year old woman in good physical condition reporting weekly physical activity such as running and muscle workout. It was her third pregnancy with two sons of 17 and 14 years old, living and free of any disease. She had a history of gestational diabetes during her second pregnancy that required insulin supplementation, but her blood sugar was well-controlled with diet and exercise for the third pregnancy. Her blood pressure and biochemistry revealed no sign of pre-eclampsia. She was referred for an ultrasound at 33 weeks to exclude foetal brain calcification because she had visited an endemic zone for Zika virus (i.e., Cuba) at 29 weeks of gestation. Blood testing for Zika at 32 weeks of gestation was negative. The father was a healthy 41-year old male with no prior children. Both parents were non-smokers and reported no familial history of genetic or neurodevelopmental disorders.

Notably, the mother reported the feeling of frequent and intense hiccups in the fetus. More precisely, from approximately 26–27 weeks of gestation, episodes of several minutes of regularly-spaced brief spasms were experienced every day (generally one or two times per day). These episodes sometimes lasted up to 20 min and occasionally featured spasms of varied intensity.

The 33 week ultrasound performed in regular clinical setting revealed the presence of multiple cardiac tumors, indicative of rhabdomyomas and of potential TSC. The mother was then referred to CHU Ste-Justine (specialized pediatric hospital) for in depth clinical investigation. Cardiac ultrasound and magnetic resonance imaging were then performed on the same day (i.e., 33 weeks and 4 days of gestation) to investigate for TSC in the fetus.

### Cardiac ultrasound

**Fig. 1:**
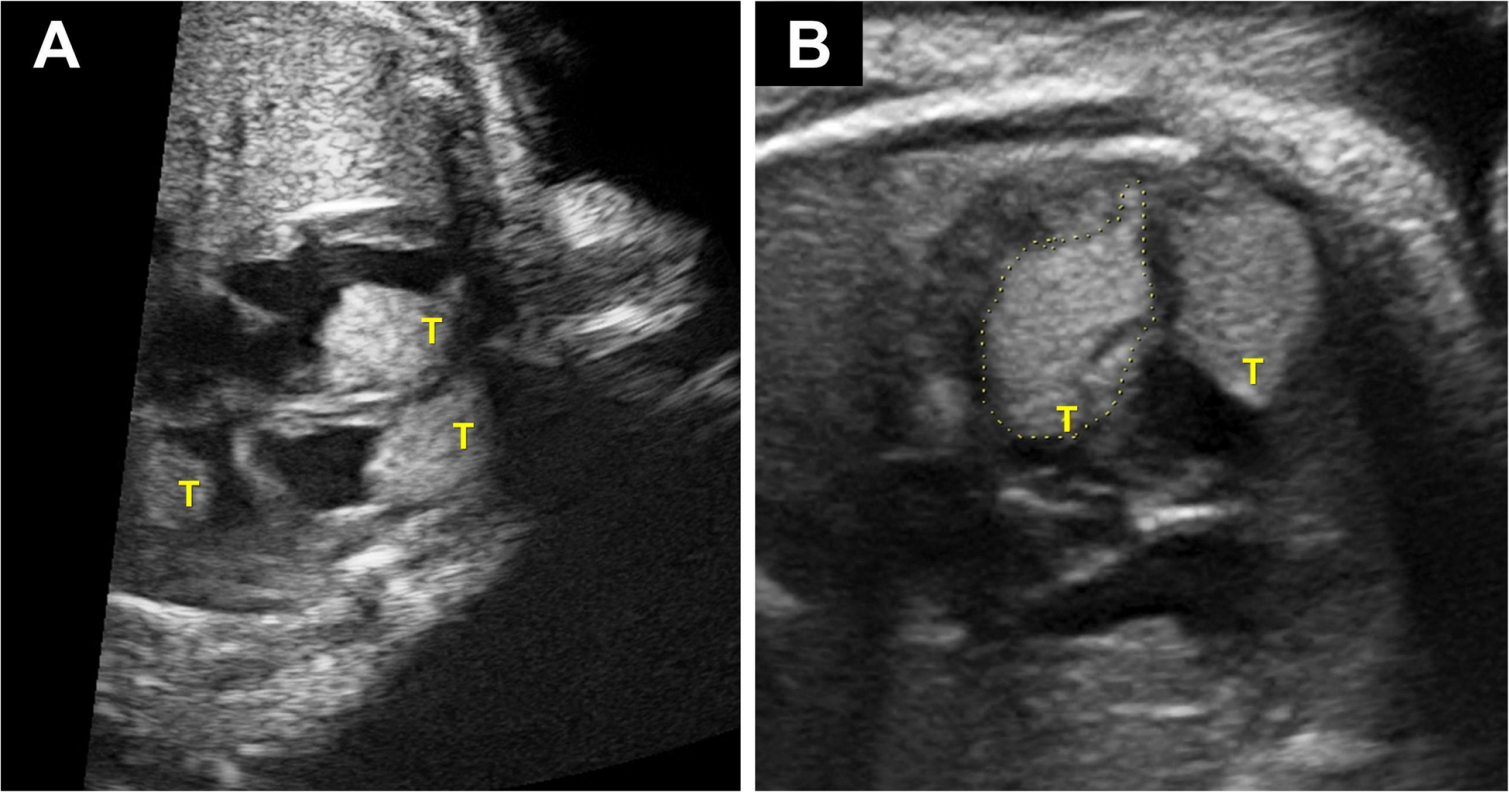
Fetal cardiac ultrasound showing the presence of tumors. **a** Image showing the two atriums and two ventricles with the presence of tumors (T) in the right atrium (observable on the left), the right ventricle as well as in the left ventricle (observables on the right). **b** Image showing tumors (T) observed in the left and right ventricles. The largest tumor (6.26 cm circumference), which was observed in the subaortic region of the left ventricle and was slightly obstructive, is surrounded by a dotted line

Cardiac ultrasound revealed the presence of at least 6 cardiac tumors in the fetus. One tumor was localized to the roof of the right atrium (Fig. 1a, left). It was a non-obstructive tumor of 3.04 cm circumference. At least two tumors were present in the left ventricle. The first was from the subaortic region to the apex and had a circumference of 6.26 cm (Fig. 1b, left). It appeared slightly obstructive of the subaortic region. The second was located posterior and at the apex, and had a circumference of 3.09 cm. At least three non-obstructive tumors were present in the right ventricle. The largest (5.37 cm circumference; Fig. 1b, right) was located in the middle, the second at the apex (3.11 cm circumference), and the third at mid-ventricular position near the septum (3.39 cm circumference). These observations are strongly indicative of multiple cardiac rhabdomyomas.

The left and right atriums were of normal size with the membrane of the oval foramen curving towards the left atrium. The left and right ventricles were also of normal size with atrioventricular and ventriculoarterial concordances. The mitral and tricuspid valves appeared normal with normal flux. The cardiac rhythm was regular, with no sign of arrhythmia. The left and right pulmonary veins appeared normally connected to the left atrium, and caval veins to the right atrium. The umbilical vein was of normal size with normal implantation of ductus venosus. The aortic valve (5.4 mm) and pulmonary valve (6.5 mm) also appeared normal. The aortic arc and isthmus appeared of good size.

Blood circulation was observed to be relatively normal. More precisely, normal flux was measured at the level of pulmonary veins and ductus venosus (S: 89 cm/s, A: 33 cm/s, pulsatility index [PI]: 0.78), at the level of mitral and tricuspid valves, as well as at the level of ascending aorta, pulmonary arteria, and arterial canal. There was a slight acceleration of the left ventricle-aorta axis (100 cm/s), likely resulting from the proximity of one of the largest tumors. Lastly, the resistance index of uterine arteries was normal (right: 0.45, left: 0.4, late systolic notch [LSN]: 0.59), and velocimetry was also normal for umbilical arteries both at the abdominal insertion (PI: 0.98, LSN: 1.3) and the placental insertion (PI: 1.13, LSN: 1.2).

### Magnetic resonance imaging

**Fig. 2:**
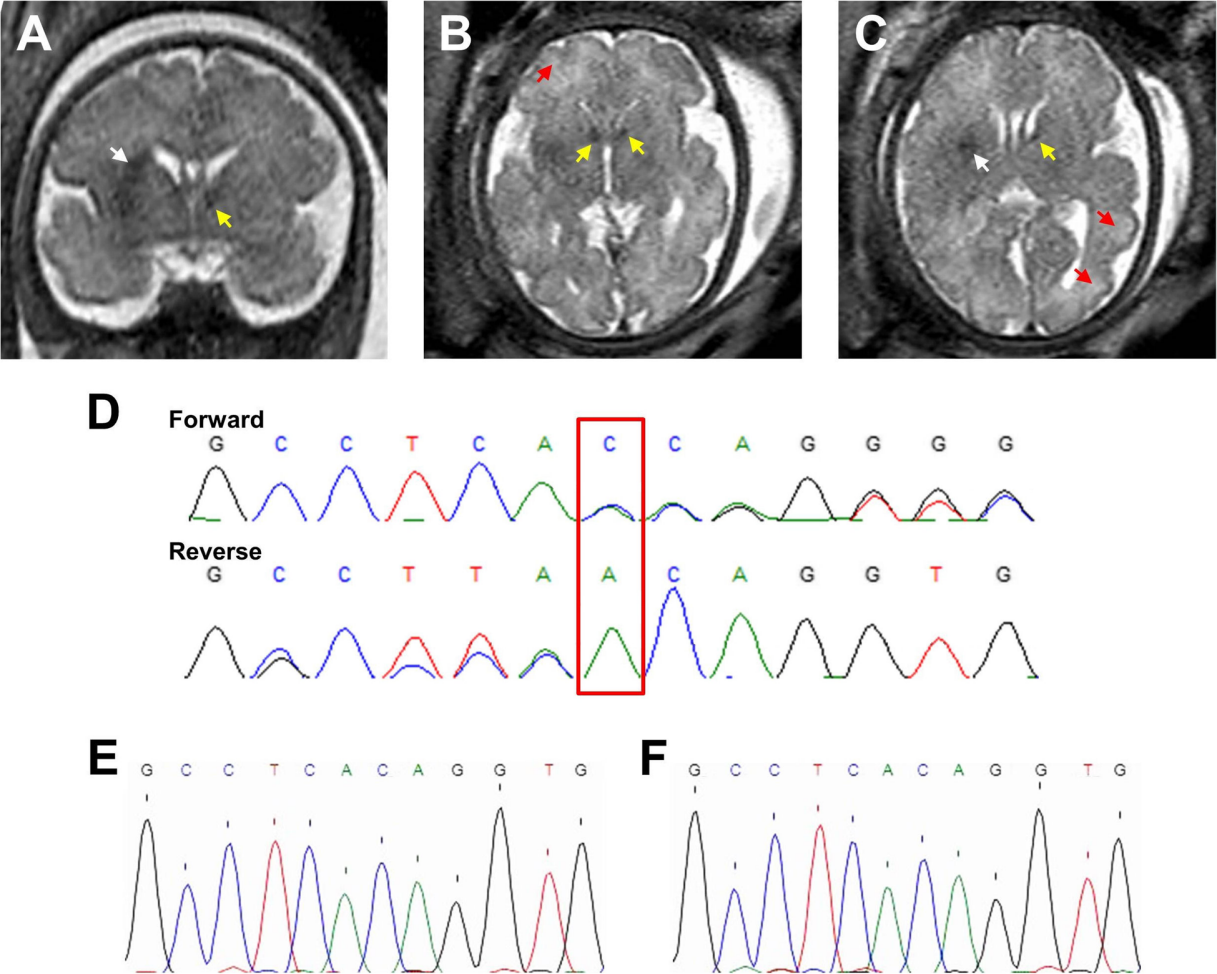
Fetal brain MRI showing nodules and hyper-intense regions, and nucleotide call from sequencing. **a** Coronal T2 image showing the presence of a sub-ependymal nodule (yellow arrow, also in **b** and **c**) and an arc-shaped white matter signal abnormality (white arrow, also in **c**). **b** Axial T2 image showing the presence sub-ependymal nodules and of a hyper-intense region (red arrow, also in **c**). **c** Axial T2 image showing a sub-ependymal nodule, a white matter signal abnormality and two hyper-intense regions compatible with the presence of cortical tubers. **d** Chromatogram and nucleotide call shown for the forward and reverse sequencing of the mutated allele only (DNA obtained from amniotic liquid) for nucleotides 5164 to 5175 of the coding sequence of *TSC2* (ENST00000219476.9). The A duplication after position 5169 is highlighted by the red box. **e** Sanger sequencing chromatogram and nucleotide call for the forward strand of the DNA amplicon obtained from the father sperm shown for *TSC2* nucleotides 5164 to 5175 of the coding sequence. No sign of A duplication was observed. **f** Sanger sequencing chromatogram and nucleotide call for the forward strand of the DNA amplicon obtained from the mother skin biopsy shown for *TSC2* nucleotides 5164 to 5175 of the coding sequence. No sign of A duplication was found

Magnetic resonance imaging (MRI) was performed the same day as the cardiac ultrasound to screen for additional markers of TSC in the fetus, with a particular focus on the central nervous system. Multiple and well-defined subependymal nodules were observed at the surrounding of both the left and right lateral brain ventricles (Fig. 2a to c). In addition, regions of abnormal signal were detected in the white matter (Fig. 2a and c) and several hyper-intense regions indicative of cortical tubers were observed throughout the cerebral cortex (Fig. 2b and c). A hyper-intense region was also localized to the left cerebellum and the possibility of cysts to kidneys was also raised.

Together, observations from the cardiac ultrasound and the MRI confirmed the diagnosis of TSC in the fetus given the finding of two of the major criteria for the disease (i.e., cardiac rhabdomyomas and subependymal nodules). The definite TSC diagnosis was further supported by additional signs of TSC (e.g., hyper-intense cortical and cerebellar regions compatible with tubers). At this stage, medical pregnancy interruption was chosen by the parents. The interruption was executed at 34 weeks and 3 days of gestation together with an amniocentesis in order to perform genetic screening (see below). The parents also requested an autopsy of the fetus, of which the main findings are reported hereafter.

### Genetic screening

The definite diagnosis of TSC strongly suggested the presence of a genetic modification in one of the two genes commonly associated with the disease, namely *TSC1* and *TSC2* [7, 9, 20]. The *TSC1* gene product is the protein hamartin and that of *TSC2* is tuberin, both of which are involved in the protein synthesis pathway [8, 27]. In general, the disease symptomatology is more severe in patients with mutations in *TSC2* compared to *TSC1* [8, 20, 21, 28]. A sequencing analysis of the *TSC1* and *TSC2* genes, including a search for duplication and deletion, was performed on the amniotic liquid sample by GeneDx (Gaithersburg, MD).

Genetic screening revealed that the fetus carried one copy (heterozygous) of a duplication of an adenine (A) at position 5169 of the coding sequence of the *TSC2* gene (*TSC2* c.5169dupA; Fig. 2d). According to genomic sequence identification number NG_005895.1 (NCBI) and transcript identification number ENST00000219476.9 (ensembl.org), this duplication occurs in exon 41 of the *TSC2* gene that contains 42 exons. The *TSC2* c.5169dupA modification is predicted to cause a frameshift starting with the codon 1724 coding for a glutamine and resulting in a change of this amino acid into a threonine and in a premature stop codon at position 5 after the reading frame is mutated (therefore denoted p.Gln1724ThrfsX5). This duplication is thus expected to be associated with nonsense-mediated mRNA decay and loss of function (or alternatively with abnormal protein function resulting from a mutated and truncated protein of 1727 amino acids instead of 1807 for the intact tuberin). Given that the c.5169dupA variant of *TSC2* is not observed in large population cohorts [29], it is considered a pathogenic variant. However, although it is recognized as being pathogenic in clinical setting, to our knowledge, no study to date has reported a causal relationship between *TSC2* c.5169dupA and TSC. No other *TSC1* and *TSC2* gene variant was detected by sequencing and deletion/duplication analysis of the fetal sample.

To verify if the genetic variant was a de novo mutation, as is the case for over two-thirds of TSC cases [8, 20], the blood of both parents was screened for the specific mutation identified in the fetus. The parents showed an absence of detectable *TSC2* c.5169dupA mutation in their blood sample. The presence of this specific mutation in the germline, specifically in paternal gametes, was further screened using DNA extraction, PCR amplification and Sanger sequencing of a sperm sample of the father (with forward primer 5′-GTTCTGGCGTGACCACCAAGTC-3′, and reverse primer 5′- CAATCTGTGCAGGGGCTTTGCTA-3′). This last genetic test revealed an absence of c.5169dupA (Fig. 2e). Given that hypomelanotic macules in TSC manifest as white skin spots, genetic testing was also performed on a biopsy of a small white skin spot (3 mm^2^) of the mother, which also revealed an absence of the fetal mutation (Fig. 2f). The absence of the fetal mutation in the blood and tissues of the parents thus supports a de novo mutation.

### Post-mortem biometry and histology

The autopsy revealed a fetus of 2556.2 g (90th percentile for 34 weeks of gestation) and 50 cm (> 90th percentile), and of 34 cm cranio-caudal length (> 90th percentile). The head circumference was 30 cm, the thorax circumference 28 cm, and that of the abdomen 24 cm. In general, biometric measurements corresponded to normal values for a gestational age of 37 weeks. The fetus did not present signs of dysmorphic features (e.g., normal ears, neck, nasal and buccal cavities, normally developed limbs). In general, the internal position and configuration of organs also appeared normal. More precisely, the left lung presented two lobes and was 24 g; the right lung presented three lobes and was 30 g; lung tissue appeared visually normal; the digestive track also appeared normal, with the liver weight of 140 g and pancreas 2.4 g. Similarly, macroscopic evaluation of the kidney, bladder and ureters revealed no abnormal position or configuration (left kidney: 14.3 g, right kidney: 14.1 g, left adrenal gland 4.5 g, right adrenal gland: 5.1 g). The same applied for the thymus, thyroid, spleen, as well as male genital organs. The results of the cardiac ultrasound were confirmed by the observation of multiple tumors in the heart.

**Fig. 3:**
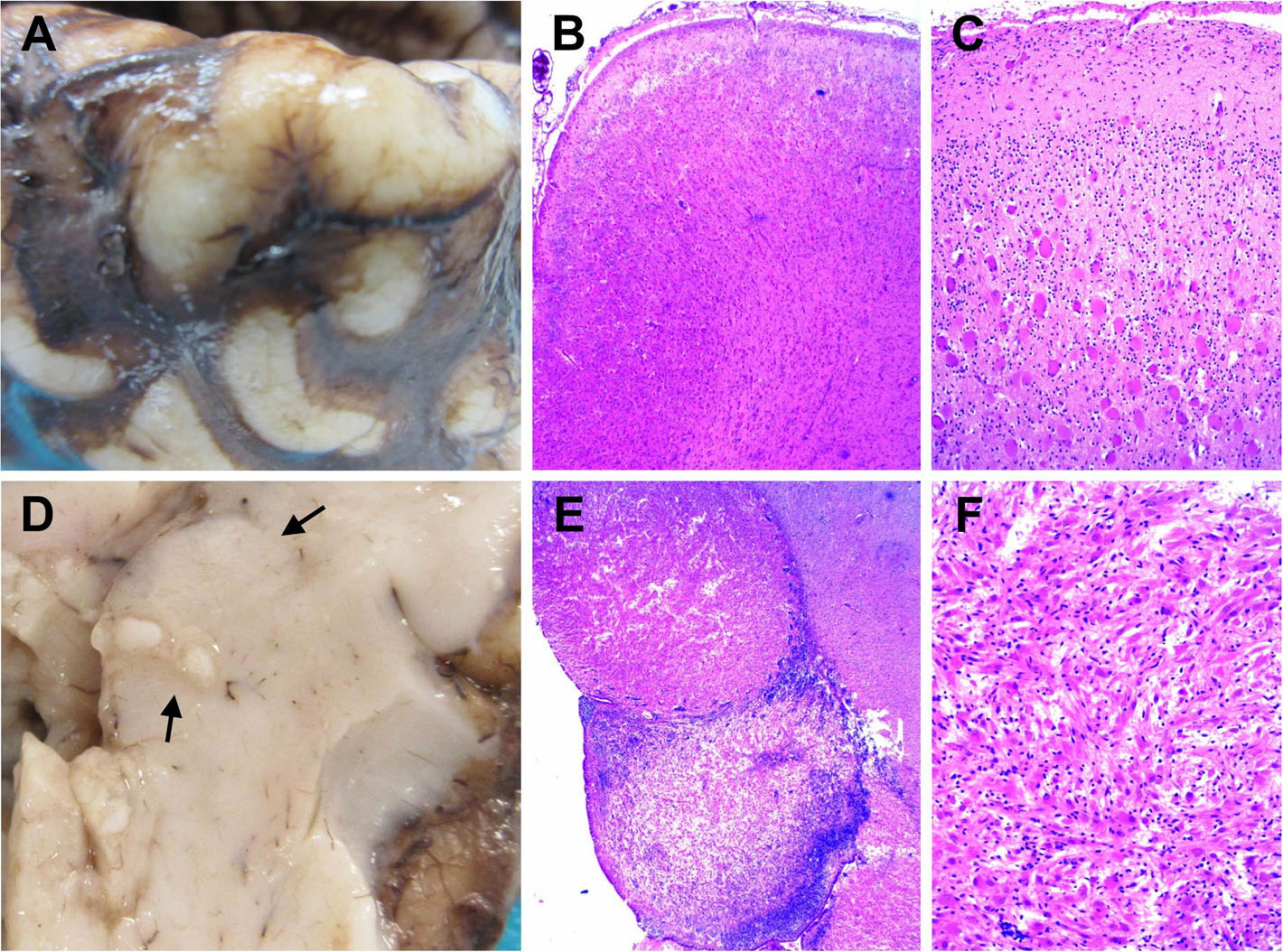
Cortical tuber and sub-ependymal nodule images and histology. **a** Widening of a gyrus caused by a cortical tuber. **b** Section of the cortical tuber showing a disorganized architecture of the cerebral cortex and the blurred margin between the grey and white matter. **c** Histologically, the cortical tuber is characterized by large abnormal astrocytic cells, sometimes with multiple nuclei, and by round “balloon cells” with a peripheral nucleus. **d** Large subependymal nodule (indicated by arrows) bulging in the ventricle. **e** Subependymal nodule located in the germinal zone, close to the head of the caudate. **f** Histologically, the subependymal nodule is made up of a mixture of elongated glial cells and some “balloon cells”

The brain weight post-fixation (formalin zinc) was 380 g. The fronto-occipital diameters were about 12.1 cm. Brain biometric measurements were corresponding to a gestational age of 37–38 weeks and were higher than the 95th percentile for a 34-week fetus. The examination of the surface of cerebral hemispheres revealed the normal presence of all primary sulci. However, some gyri appeared to have a pachygyric aspect, with a firm consistency upon touch suggesting the presence of cortical tubers (Fig. 3a). These firm areas were noted to be predominant in the left hemisphere, particularly in the left frontal lobe. The external morphology of the spinal cord appeared normal.

The examination of coronal slices of cerebral hemispheres revealed areas of firmer consistency in the white matter and at the hemispheric surface. Microscopic examination exposed the presence of diffuse lesions of the white matter composed of globular astrocytes and organized in track alongside the axis of vessels. These lesions were associated with typical “balloon cells” and were abundant and extended, appearing in almost all cerebral lobes and sometimes almost occupying an entire gyrus. When they involved the cerebral cortex, they were responsible for a disorganization of the cortical cytoarchitecture (Fig. 3b). Small and numerous cortical lesions (tubers) were observed at the surface of the cortex, sometimes isolated but most often in continuity with white matter lesions described above (Fig. 3a and b). These were characterized by large abnormal astrocytic cells and round “balloon cells” (Fig. 3c). Astrocytes of white matter lesions and tubers strongly expressed the protein GFAP (glial fibrillary acidic protein), which was not the case for nearby balloon cells. These lesions were also accompanied with microglial activation (sometimes particularly marked). Brain ventricles did not appear to be enlarged but numerous bilateral subependymal nodules were observed in lateral ventricles (Fig. 3d). Nodules were found in the germinal zone, near the sulcus terminalis, in anterior, near the head of the caudate nucleus (Fig. 3e), and in posterior, close to the thalamus. They were made up of large astrocytes with elongated cytoplasm (Fig. 3f), showing a weak GFAP staining. The examination of axial slices of the mesencephalon showed a very narrow aqueduct of Sylvius. One small lesion, suggestive of a tuber, was also found in the cerebellum, of which the parenchyma was intensely congested. No calcification was observed at this prenatal/precocious stage. The multiplicity and extent of cortical lesions and subependymal nodules observed in the fetus supported a particularly severe form of TSC.

## Discussion and conclusions

In this report, a particularly severe case of TSC was associated with a pathogenic mutation in the *TSC2* gene. Firstly, a definite diagnosis of TSC was made in utero with observations of multiple cardiac rhabdomyomas and subependymal nodules using cardiac ultrasound and MRI at gestational age 33 weeks. Secondly, genetic testing revealed a duplication in exon 41 of the gene coding for the protein tuberin, predicted to change and truncate the reading frame. Thirdly, a high severity of brain damage was established with brain histology revealing widespread lesions in both the white and grey matter of the cerebrum together with multiple subependymal nodules. Together, these observations stress the importance of multi-organ investigation in prenatal diagnosis of TSC, support the literature linking *TSC2* mutations to a higher disease severity, and can assist the establishment of a prognosis and of a treatment strategy in the context of prenatal TSC diagnosis.

The present observations highlight the importance of a third trimester (here 33 weeks) ultrasound during pregnancy to detect neurodevelopmental diseases. A third trimester ultrasound investigation is rarely implemented as a routine examination in clinical settings although it has a definite value for early diagnosis and intervention [30], especially in the context of TSC [26, 31]. Nonetheless, in some countries, this specific examination seems more common given the relatively frequent occurrence of TSC diagnosis in utero [31]. An early diagnosis of TSC is critical for the establishment of both an optimal monitoring (comprising regular electroencephalography) and a treatment strategy [31], which can benefit cognitive outcomes when implemented before seizure onset [32]. In the present severe case, the choice of medical pregnancy interruption, which is legal in Canada, was permitted by the third trimester ultrasound.

In addition, this report provides support for the systematic use of investigation with multiple modalities in the context of fetal diagnosis of TSC, in particular concerning the combination of ultrasound and MRI (and of genetics). This is in line with recommendations made by others with regards to the value of multi-organ investigation in TSC and that of fetal MRI [26, 31, 33]. Moreover, using this combined set of tools may also eventually serve to inform parents of the (high) likelihood of severe disease. Even if it remains unclear whether TSC severity is higher when brain lesions are observed in utero [26], in the present case, the simultaneous observation of multiple features of the disease (many large rhabdomyomas, numerous bilateral subependymal nodules, abnormal white matter signal, hyper-intense cortical and cerebellar regions) not only enabled a definite diagnosis but also suggested a more severe form of the disease. Given that the extent of brain lesions in TSC generally correlates with neurological and psychiatric symptoms [7, 17, 18, 34], and that it is also the case for lesions in other organs such as cardiac rhabdomyomas [5], inference on prognosis can (and should) be made. In the future, refinement of fetal MRI may also allow improved quantitative and qualitative measurements of cortical lesions that will importantly inform prognosis given that, for instance, global tuber load and the presence of cyst-like tubers can be indicative of more severe infantile spasms and epilepsy [34, 35].

The high severity of the present TSC case was also strongly supported by genetic testing and histology. Indeed, the identification of a duplication in the *TSC2* gene in the fetus having multiple and large lesions is in agreement with a more severe symptomatology generally observed in patients with *TSC2* mutations in comparison to patients with *TSC1* mutations or no identified mutation [8, 9, 20, 21, 28]. The fact that the parents did not show the presence of the mutation is also supportive of a more severe disease in cases of sporadic *TSC2* mutations [9]. As performed for other genetic variants related to TSC [21, 24], functional studies are now required to investigate whether *TSC2* c.5169dupA leads to an altered mRNA processing, an abnormal protein function or a loss of function. *TSC2* c.5169dupA is not listed in the Leiden Open Variation Database (LOVD, http://www.lovd.nl/TSC2) [36] and is therefore a novel mutation, similar to those described in a recent case report [37]. However, c.5169dupA generates a modification of Gln1724, which is reported a few times in the LOVD. Together, these observations point to a key genomic region of *TSC2* in the context of TSC. Given the potential for severity of TSC, even if precise *TSC1* and *TSC2* mutations are rarely associated with well-defined phenotypes [9, 22, 23], there is a need to systematically inform future parents about the disease and to offer early pregnancy genetic testing (at least for pathological mutations that have been associated to severe TSC), similar to what is currently offered for Down syndrome.

Infantile spasms cannot be ascertained during the fetal period due to the impossibility to record the fetus electroencephalogram. Nevertheless, given the association between early onset epilepsy/infantile spasms and disease severity [23, 27], developing tools to investigate early signs of infantile spasms during pregnancy is likely important to complement the evaluation of disease severity and to better inform parents. In the present case, the mother had reported a potential additional sign of TSC during pregnancy, in the presence of frequent and intense spasm episodes in the fetus described as a hiccup sensation. Although there is actually no evidence for this particular report to be indicative of infantile spasms, more attention to this type of anecdotal observation and its association with cardiac and brain lesions in the context of the diagnosis of TSC during pregnancy seems relevant. In fact, clinical research is required to determine whether the interrogation of the mother about fetal hiccups/spasms may assist TSC diagnosis or the evaluation of TSC severity.

In general, better education of the population and especially of future mothers about this rare genetic disease is important to minimize psychological distress upon TSC diagnosis in the progeny. The present report emphasizes the importance of a detailed multi-organ investigation accompanied by genetic testing in the context of TSC diagnosis during the fetal period. It also supports the evaluation of TSC severity using the same tool set. Severity assessment is tremendously important in guiding the parents in the context of medical pregnancy interruption as well as regarding the suitability of early treatment. The use of mTOR inhibitors is generally well-tolerated in patients, including infants, and has been associated with clinical improvements in young TSC patients with different types of tumors [38–41]. For instance, everolimus was shown to reduce the size of cardiac tumors and brain abnormalities in neonates [40, 41], and could be used in similar cases of apparent severe TSC. Such cases could also benefit from early interventions with antiepileptic drugs [32] and even from treatment of the mother in the course of pregnancy after TSC diagnosis in utero, in order to prevent/diminish some manifestations of the disease.

## Data Availability

The raw acquisitions used and analysed in the context of the current Case report are available from corresponding authors on reasonable request.

## Abbreviations

CHU: Centre hospitalier universitaire
GFAP: Glial fibrillary acidic protein
LSN: Late systolic notch
MRI: Magnetic resonance imaging
mRNA: Messenger ribonucleic acid
PI: Pulsatility index
TSC: Tuberous sclerosis complex
*TSC1*: Tuberous sclerosis complex 1
*TSC2*: Tuberous sclerosis complex 2
